# Lyme disease in UK primary care: a knowledge, attitude, and practice survey

**DOI:** 10.3399/BJGPO.2024.0092

**Published:** 2025-03-12

**Authors:** Lucy Delaney, Amanda Semper, Neil French, John SP Tulloch

**Affiliations:** 1 NIHR Health Protection Research Unit in Emerging and Zoonotic Infections, University of Liverpool, Liverpool, UK; 2 Institute of Infection, Veterinary and Ecological Sciences, University of Liverpool, Liverpool, UK; 3 Rare and Imported Pathogens Laboratory, UK Health Security Agency, Porton Down, UK; 4 Institute of Infection, Veterinary and Ecological Sciences, University of Liverpool, School of Veterinary Science, Neston, UK

**Keywords:** Diagnosis, health knowledge, attitudes, practice, Lyme disease, primary care

## Abstract

**Background:**

Lyme disease (LD) cases in the UK most commonly present within the primary care setting. Despite an upward trend of incidence, little is known regarding GP experience with diagnosis and treatment.

**Aim:**

This study aims to describe baseline primary care clinician Knowledge, Attitude and Practice (KAP) in Scotland and England.

**Design & setting:**

Online KAP survey on LD for UK-based practising GPs.

**Method:**

An online KAP questionnaire was developed for use in UK primary care. The survey was distributed through UK-based research networks, professional societies, and via social media.

**Results:**

A total of 191 complete responses were analysed (England *n* = 130, Scotland *n* = 61). The Scotland-based responder group had more relevant consultations in the previous 3 years. Responders from Scotland demonstrated a greater awareness that erythema migrans (EM) is pathognomonic for LD and that serological testing of this patient group is not indicated. Less common cardiac and neurological symptoms were not as well associated with LD by both responder groups for the former and England-based responders for the latter. Prescribing according to the National institute for Health and Care Excellence (NICE) guidance was identified in 70% of Scotland and 42% of England-based GP responses.

**Conclusion:**

Targeted resources may improve clinician confidence on exposure risk, symptom recognition, testing limitations and treatment dose and duration. Scotland-based responders’ better survey performance potentially reflects greater clinical exposure and public awareness of the disease, due to high endemicity within the nation.

## How this fits in

National Institute for Health and Care Excellence (NICE) Lyme disease (LD) guidance was introduced in 2018, in the wake of increasing media attention and patient advocacy. Most LD patients will present in the primary care setting, but little is known regarding GP awareness of the disease and patient management. This novel KAP survey will provide baseline data on current GP practice and their interpretation and implementation of NICE guidance. Comparison of nations with differing incidence will identify whether implementation of NICE guidance varies with clinical exposure.

## Introduction

LD is endemic within the UK and is the most common tick-borne infection in the northern hemisphere, caused by transmission of bacteria of the *Borrelia burgdorferi sensu lato* complex, via the bite of an infected sheep tick (*Ixodes ricinus*).^1–3^

There has been an upward trajectory of LD incidence over the previous two decades. UK incidence data is based on passive surveillance, through clinical requests for investigation, largely based on serologically confirmed cases using two-tiered serological testing methodology.^4–7^ Incidence is highly variable between nations and regionally. National incidence in Scotland and England is 6.8 and 2.0 cases per 100 000 respectively, with the highest regional incidence (44.1 cases per 100 000) in the Scottish Highlands.^5–7^ Laboratory-confirmed incidence may be influenced through local referral patterns and does not include clinically diagnosed cases, with the true LD burden estimated to be between two to over fivefold greater.^8–11^

Early localised infection is characterised by an erythema migrans (EM) rash, spreading from the tick bite site from 3 to 30 days after infection, but is not always observed and may vary in appearance.^1,9^ EM is diagnostic for LD with recommendation to treat without further confirmatory testing.^1,12^ Prompt antibiotic treatment can prevent disseminated and late disease stages, avoiding potential neurological, musculoskeletal, cutaneous, and cardiac complications.^1,13^ The non-specific nature of LD symptoms can complicate diagnosis in the absence of EM rash or known tick exposure.^14,15^ Laboratory confirmation can be useful in such cases, but is often negative during early infection. Due to antibody persistence, positive serological results do not necessarily reflect active infection.^12^

Clinician awareness and patient management may be influenced by local endemicity and public health initiatives, as well as clinical experience of LD.^16,17^ UK educational resources for clinicians include the 2018 NICE guidelines and the Royal College of General Practitioners (RCGP) e-learning module, providing comprehensive guidance to standardise patient diagnosis and management.^12,18^ There is limited UK evidence regarding clinician awareness and practice. KAP surveys provide a time and cost-efficient means to capture relevant quantitative and qualitative data, of value for identifying and measuring change in KAP and validating LD focused initiatives.^19–22^ We used a GP KAP survey relevant to LD in the UK, to assess GP awareness and practice in relation to NICE guidance. Responses from GPs in Scotland and England were compared, to identify whether there were differences in KAP between nations of high and lower incidence.

## Method

An online GP KAP survey was developed, adapting a previously validated questionnaire from the US and Canada for UK relevance.^16,20–22^ The survey was designed online (JISC surveys), with the final version containing 28 question sets and provision of exemplar responses based on NICE guidance, on response submission (Supplementary Box 1).

Pilot testing by five local GPs and two volunteers from the Lyme Disease Action charity, generated feedback on question/response structure and completion time.

Dissemination of the survey link was attempted through: English Clinical Research Networks, Scottish Primary Care Research Networks, social media, the Primary Care Dermatology Society (PCDS) and the RCGP. Circulation commenced on 10 June 2022 and survey access was closed on the 10 March 2023.

Responder demographics were described and mapped to nation of practice via postcode area. Representativity of responses by nation, was assessed by comparing response data with national GP workforce data from 2022,^23,24^ stratified by age distribution and sex, using χ^²^ tests.

Responder groups from Scotland and England were compared using χ^²^ tests, for NICE guidance related questions with a single correct response. ‘Don’t know’ responses were allocated to the incorrect response option. Free text responses were thematically grouped for qualitative analysis, with more than one theme possible per response. Themes were expanded as necessary to accommodate all responses.

All statistical analysis was performed using Open Source Epidemiologic Statistics for Public Health (OpenEpi, version 3.01).

## Results

**Figure 1.**
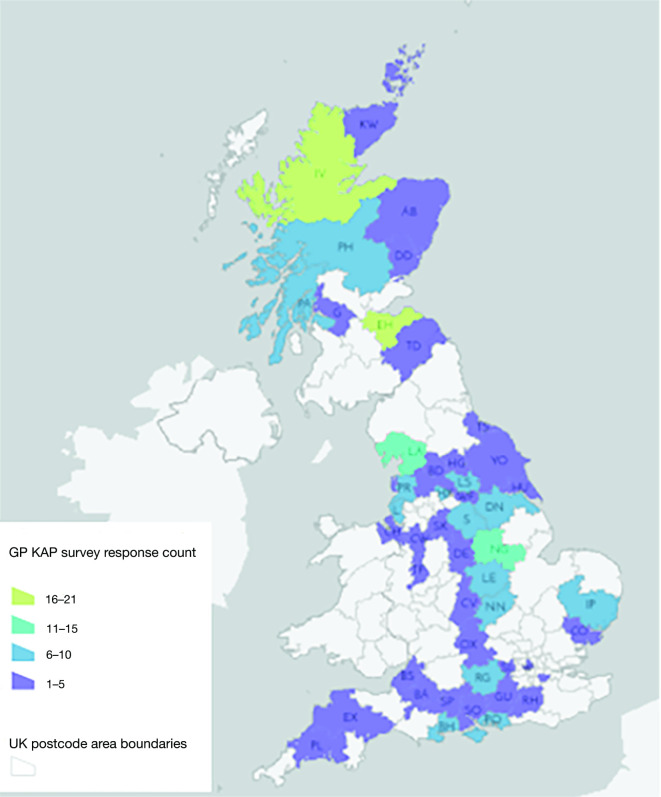
GP LD KAP survey response count by responder UK postcode area

A total of 193 completed surveys were received, with 191 (*n* = 60 Scotland and *n* = 131 England) included in the final analysis. A single response from Wales and Northern Ireland were excluded, due to lack of responder representation for these nations. ‘Don’t know‘ or equivalent responses were recorded for 8% and 12% of questions in Scotland and England-based responders respectively (Supplementary Table 1). Response count was mapped by postal area (Figure 1).

Survey links were accessed 1174 times, giving an overall response rate of 16% (Table 1).

**Table 1.** Survey response count and response rate by circulation route

	CRN (England)	SPCRN (Scotland)	Social media	PCDS	RCGP	Total
Survey access count, *n*	326	211	609	16	12	1174
Response rate, %	33.1	25.1	5.1	6.3	0	16.4

CRN = Clinical Research Network. PCDS = The Primary Care Dermatology Society. RCGP (Royal College of General Practitioners. SPCRN = Scottish Primary Care Research Network.

Age band data was too sparse for statistical analysis, although over 60% of responders in both nation groups were between 31–50 years of age, in line with 2022 national GP data (Table 2). Female responders were overrepresented in the Scotland-based group when compared to national data from 2022 (*P* = 0.02), but not in the England-based responder group (*P* = 0.24).

**Table 2.** Survey participant demographics and LD relevant consultations by nation

Demographic		Scotland survey (*N* = 61), % (95% CI)	Scottish national, %	*P*-value	England survey (*N*=130), % (95% CI)	English national, %	*P*-value	Odds ratio (95% CI)
**Age band, years**								
<31		4.9	0.6	n/a	0.8	0.6	n/a	n/a
31-40		29.5	30.6	n/a	23.1	26.7	n/a	n/a
41-50		36.1	34.3	n/a	40.8	34.9	n/a	n/a
51-60		26.2	28.9	n/a	30.0	27.7	n/a	n/a
>60		3.3	5.6	n/a	5.4	10.1	n/a	n/a
**Sex**								
Female		73.8	61.6	0.02	53.8	56.9	0.24	n/a
**Relevant patient consultations in previous 3 years**								
Tick bite		96.7 (88.65 to 99.6)	n/a	n/a	80.0 (72.08 to 86.5)	n/a	<0.001	7.38 (1.69 to 32.18)
Suspected LD		93.4 (84.05 to 98.18)	n/a	n/a	70.8 (62.15 to 78.41)	n/a	<0.001	5.89 (2.0 to 17.36)
Treated LD		91.8 (81.9 to 97.28)	n/a	n/a	63.8 (54.96 to 72.08)	n/a	<0.001	6.34 (2.38 to 16.94)
**Practice catchment area (multi-response)**								
Rural		59.0 (45.68 to 71.45)	n/a	n/a	23.8 (16.81 to 32.11)	n/a	<0.001	4.60 (2.4 to 8.81)
Urban		31.1 (19.90 to 44.29)	n/a	n/a	40.8 (32.24 to 49.73)	n/a	0.10	0.66 (0.34 to 1.25)
Suburban		11.5 (4.74 to 22.23)	n/a	n/a	36.2 (27.92 to 45.04)	n/a	<0.001	0.23 (0.09 to 0.54)

n/a = value or calculation not applicable. CI = confidence interval. LD = Lyme disease.

Almost all responders worked in NHS GP practices, with some additional out of hours/urgent treatment centre roles (Supplementary Table 2). Scotland-based responders more commonly identified their practice catchment area as rural (odds ratio [OR] = 4.60, 95% confidence interval [CI] = 2.4 to 8.81) and were more likely to indicate LD relevant consultations in the previous 3 years (Table 2).

Additional demographic data provided in Supplementary Table 2.

### Geographical knowledge

**Figure 2.**
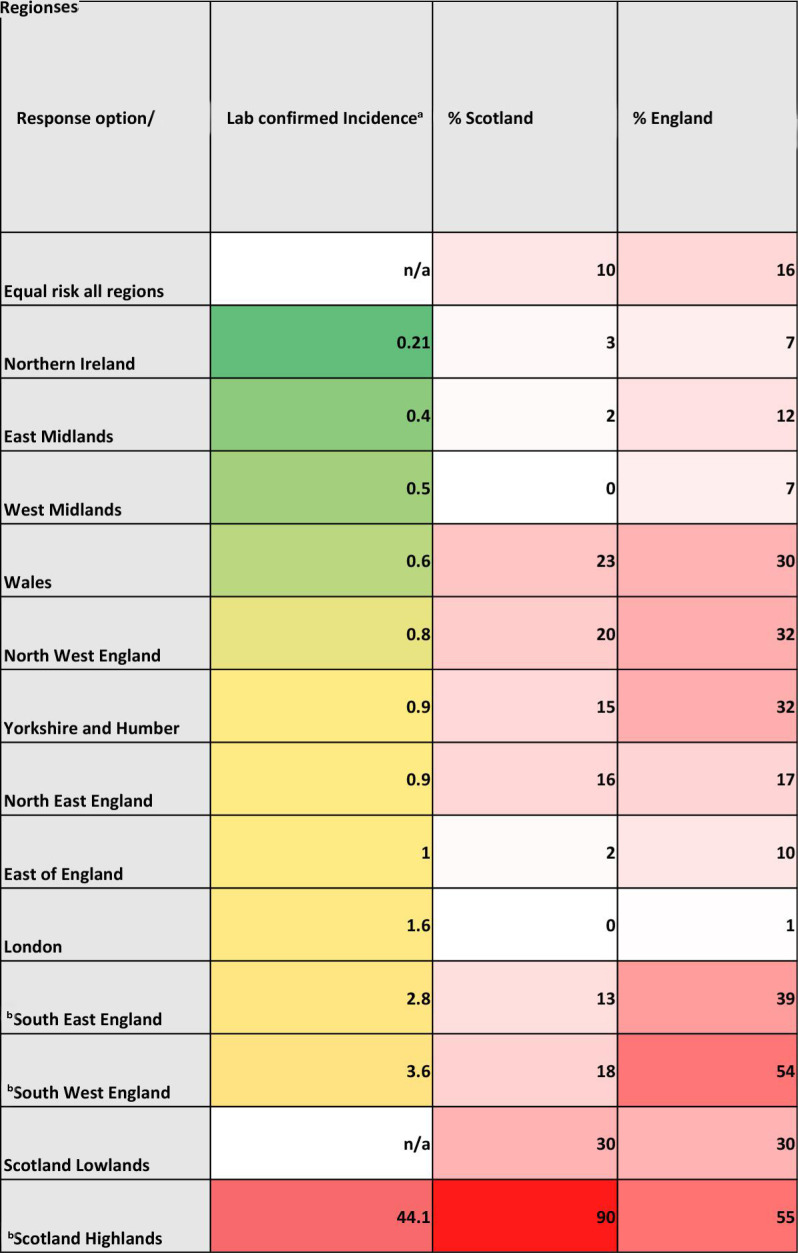
Percentage of responders (by nation), who indicated that they perceived listed regions to be high-risk for LD, alongside published regional laboratory confirmed incidence rates (cases per 100 000). ^a^Estimated published laboratory confirmed incidence figures (cases per 100 000).^5,6,25,26^ ^b^Denoted as high-risk areas in NICE guidance.^12^

The higher risk of contracting LD in the Scottish Highlands and Islands was recognised by 90% of Scotland-based responders and 55% of England-based responders. The England high-risk regions in the South West and South East, were less well recognised by Scotland-based responders. Other nations and regions showed varying responses that did not align with laboratory incidence rates (Figure 2).^5,6,25,26^

Just over half of responders recognised that tick habitat may occur in urban and suburban green space, as well as rural areas (Table 3). Further stratifying nations by practice catchment area type showed no statistically significant difference (Supplementary Table 3).

**Table 3.** Knowledge-based questions with percentage stated response by nation

Survey response	Scotland response (*N* = 61), % (95 CI)	England response (*N* = 130), % (95 CI)	OR (95% CI)	*P*-value
**Tick exposure**				
Infected ticks may occur in green space in: (rural, suburban and urban response)	55.7 (42.45 to 68.45)	44.6 (35.9 to 53.58)	1.56 (0.85 to 2.88)	0.08
**Diagnosis (True/False): correct response score**				
*False*: Tick bite recall is necessary for clinical suspicion of LD	96.7 (88.65 to 99.6)	94.6 (89.22 to 97.81)	1.68 (0.34 to 8.33)	0.28
*False*: EM appears within ≤24 hours of a tick bite	88.5 (77.77 to 95.26)	70.0 (61.34 to 77.72)	6.23 (1.41 to 27.62)	<0.01
*True*: EM is an expanding lesion with a partial central clearing	100 (94.13 to 100)	96.9 (92.31 to 99.16)	n/a	n/a
*True*: EM is sufficient for a clinical diagnosis of LD	93.4 (84.05 to 98.18)	76.2 (67.89 to 83.19)	3.89 (1.29 to 11.67)	<0.01
*False*: EM is always present in LD	96.7 (88.65 to 99.6)	93.1 (87.26 to 96.79)	0.98 (0.09 to 10.97)	0.47
*True:* Multiple EM may be on different areas of the body	39.3 (27.07 to 52.69)	45.4 (36.64 to 54.35)	0.74 (0.38 to 1.44)	0.19
*True:* EM may be located in a different area from tick bite	63.9 (50.63 to 75.84)	54.6 (45.65 to 63.36)	1.54 (0.76 to –3.12)	0.12
**LD laboratory testing (True/False): correct response score**				
*False*: A patient with EM should be tested for LD	63.9 (50.63 to 75.84)	26.9 (19.52 to 35.4)	4.81 (2.51 to 9.22)	<0.001
**LD associated signs and symptoms (multi-response)**				
Fatigue/lethargy	100	100	^a^	^a^
Diffuse myalgia and arthralgia	100	99.2	^a^	^a^
EM	100	98.5	^a^	^a^
Fever	93.4	90.0	^a^	^a^
Arthritis	91.8	92.3	^a^	^a^
Cranial neuritis ± facial nerve palsy	93.4	68.5	^a^	^a^
Atrioventricular block	57.4	55.4	^a^	^a^
Provided all 7 valid responses above	55.7	47.7	^a^	^a^
**Treatment (adult without focal symptoms — first choice treatment)**				
Doxycycline	98.4 (91.2 to 99.96)	86.2 (79 to 91.58)	9.64 (1.26 to 74)	<0.01
Duration of 21 days	83.3 (71.48 to 91.71)	57.1 (47.45 to 66.45)	3.75 (1.73 to 8.14)	<0.001
Dose of 200 mg daily	80.0 (67.67 to 89.22)	56.3 (46.56 to 65.61)	3.11 (1.49 to 6.49)	<0.001
Correct dose and duration	70.0 (56.79 to 81.16)	42.0 (32.7 to 51.66)	3.23 (1.66 to 6.29)	<0.001

True and False response options: analysis included a ’don’t know’ response with the incorrect response option. If applicable, ’don’t know’ responses (%) for each responder group are tabulated in Supplementary Table 1. ^a^Multi-response options may have values >100%, with no associated OR or *P*-value calculable. CI = confidence interval. EM = erythema migrans. LD = Lyme disease. n/a = not applicable. OR = odds ratio.

### Knowledge response analysis

Strategies to prevent tick bites were well recognised, although use of a DEET containing insect repellent and wearing light-coloured clothing were less well known (Supplementary Table 4). *B. burgdorferi sensu lato* was identified as a bacteria by the majority of responders.

Most responders were aware that lack of patient recall of a tick bite should not preclude a LD diagnosis, along with recognition of a description of an EM rash and that EM is not observed in all cases of LD (Table 3). Scotland-based responders were more likely to differentiate between EM and an early tick bite reaction (OR 6.23, 95% CI = 1.41 to 27.62) and were more likely to recognise that EM is diagnostic for LD (OR 3.89, 95% CI = 1.29 to 11.67). They were also more aware that this patient group does not require further laboratory testing (OR 4.81, 95% CI = 2.51 to 9.22). England-based responders also less commonly associated cranial neuritis (with or without facial nerve palsy (FNP)) with LD (93.4% of Scotland-based responders versus 68.5% of England-based responders). All responders were less likely to recognise atrioventricular (AV) block and disseminated EM symptoms of multiple rashes or a rash sited away from recalled tick bite.

Scotland-based responders were more likely to prescribe doxycycline as a first-choice treatment for an adult with LD without focal symptoms (OR 9.64, 95% CI = 1.26 to 74). Overall, 70.0% of Scotland-based responders compared to 42.0% of England-based responders would prescribe doxycycline at the NICE guidance recommended dose of 200 mg daily (OR 3.11, 95% CI = 1.49 to 6.49) and 21-day duration (OR 3.75, 95% CI = 1.73 to 8.14). This difference between nations remained when further stratified by practice catchment area type, although improved prescribing to NICE guidance within rural UK settings may be inferred (Supplementary Table 3).

Additional Knowledge response data provided in Supplementary Table 4.

### Attitude responses

Most responders considered that LD occurred within their practice area and disagreed with the statement that LD is a likely outcome if bitten by a tick in the UK (Table 4).

**Table 4.** Attitude and Practice questions with percentage response by nation

Survey response	Scotland response (*N* = 61), % (95% CI)	England response (*N* = 130), % (95% CI)	OR (95% CI)	*P*-value
**Attitudes responses**				
Disagreed ‘LD is a likely outcome if a person is bitten by a tick in the UK‘	86.9 (75.78 to 94.16	81.5 (73.79 to 87.80)	1.92 (0.74 to 5.00)	0.09
Considers LD to occur within their practice region	93.4 (84.05 to 98.18)	86.9 (79.89 to 92.19)	2.14 (0.69 to 6.67)	0.09
**Practice/clinical scenario**				
Consultation with asymptomatic patient with attached and engorged tick				
Advise/remove tick	100 (94.13 to 100)	95.4 (90.22 to 98.29)	^a^	^a^
Don't know response	0	3.1 (0.84 to 7.69)	^a^	^a^
Other response	0	1.5 (0.19 to 5.45)	^a^	^a^
Indicated method of tick removal (multi-response)				
Tick removal tool	96.7 (88.65 to 99.6)	89.2 (82.59 to 93.99)	^a^	^a^
Further advice (themes from free text responses)				
No further advice indicated	18.0 (9.36 to 29.98)	14.6 (9.03 to 21.88)	^a^	^a^
Prophylaxis	8.2 (2.72 to 18.1)	26.2 (18.84 to 34.58)	^a^	^a^
Symptoms advice — EM alone	24.6 (14.46 to 37.29)	22.3 (15.48 to 30.44)	^a^	^a^
Symptoms advice — EM and non-EM rash symptoms	55.7 (42.45 to 68.45)	40.8 (32.24 to 49.73)	^a^	^a^
Treating a patient with a positive non-NHS approved LD test				
Would treat patient on basis of test	18.0 (9.36 to 29.98)	35.4 (27.2 to 44.25)	^a^	^a^
Would not treat patient on basis of test	23.0 (13.15 to 35.5)	11.5 (6.60 to 18.32)	^a^	^a^
Would consult guidance	41.0 (28.55 to 54.32)	47.7 (38.86 to 56.63)	^a^	^a^
Other (free text response see Table S4a)	18.0 (9.36 to 29.98)	5.4 (2.19 to 10.78)	^a^	^a^
Clinically manage a patient with ongoing symptoms of headache and fatigue following two courses of antibiotics for LD with EM				
Refer to an Infectious Disease specialist	95.1 (86.29 to 98.97)	72.3 (63.78 to 79.79)	^a^	^a^
Discuss other possible causes for symptoms	55.7 (42.45 to 68.45)	57.7 (48.72 to 66.3)	^a^	^a^
Discuss symptoms may not be active disease — keep under review	31.1 (19.9 to 44.29)	31.5 (23.67 to 40.27)	^a^	^a^
Refer to other specialist	8.2 (2.72 to 18.1)	17.7 (11.56 to 25.35)	^a^	^a^
Prescribe a third course of antibiotics for LD	4.9 (1.03 to 13.71)	1.5 (0.19 to 5.45)	^a^	^a^

True and False response options: analysis included a ’don’t know’ response with the incorrect response option. If applicable, ’don’t know’ responses (%) for each responder group are tabulated in Supplementary Table 1. ^a^Multi-response options may have values >100%, with no associated OR or *P*-value calculable. CI = confidence interval. EM = erythema migrans. LD = Lyme disease. OR = odds ratio.

### Practice and clinical scenario responses

#### Asymptomatic patient with an attached engorged tick

The majority of responders indicated that they would remove the tick, most commonly using a tick removal tool. Free text themes identified further advice given to the patient, with 20% recommending observing for emerging EM only. Consideration of prophylaxis was more common in England-based responses (8% Scotland, 26% England).

#### A patient with a positive LD test from a non-NHS approved source

Almost half of responders would consult current guidance due to uncertainty in this scenario. England-based responders would more commonly consider treating (18% Scotland, 35% England) than not treating the patient (23% Scotland, 12% England) based on a non-NHS approved test. Free text themes included clinical context with patient symptoms; NHS laboratory retest; laboratory and test credentials; Clinical history and serological details (Supplementary Table 5).

#### Post-treatment symptoms of headache and fatigue after two courses of antibiotics

Referral to an infectious disease specialist was most frequently indicated by responders (95% Scotland, 72% England), with over half of responders discussing other possible causes of symptoms. A minority would refer to other specialists (8% Scotland, 18% England). Over 30% would keep the patient under review. A minority indicated that they would prescribe a third dose of antibiotics.

Additional attitude and practice response data provided in Supplementary Table 5.

## Discussion

### Summary

This survey provides baseline UK relevant KAP data, with Scotland-based responders more commonly adhering to NICE guidance in respect of diagnostic criteria and prescribing. Patient management may be influenced through inappropriate test referrals for patients with EM, which was the lowest correct scoring knowledge question for England-based responders. Less than half of this responder group were aware of NICE recommended prescribed dose and/or duration for doxycycline. GP responders were knowledgeable on many LD-associated symptoms and there was good awareness that a lack of patient tick bite recall, or observable EM, should not preclude a LD diagnosis. Improving clinician awareness on lower scoring KAP items may reduce diagnostic uncertainty and improve patient confidence, as well as reducing the potential for treatment delay or misdiagnosis.

### Strengths and limitations

As far as we are aware, this is the first attempt to describe GP LD KAP within the UK. By grouping responders from a high and lower incidence nation, we have identified areas of KAP that may reflect levels of clinical experience of LD and may be influenced through national or regional LD initiatives. Educational resources and future signposting may be targeted and validated by measuring changes to KAP over time.

Opportunities to include all UK nations were missed, due to the negligible response counts from Wales and Northern Ireland and predictably, the survey response rate was low.^27^ Many responders indicated relevant LD consultations, possibly reflecting a more invested GP group with greater clinical experience, further limiting generalisability to the UK GP population. Data on consult rates and improved regional coverage, would have proved useful to infer clinical experience and stratify at a regional level. Risk factors for KAP scores are likely to be multi-factorial and may be influenced through differences in devolved healthcare systems and regional initiatives, which were beyond the scope of this KAP study. The survey was online, providing an opportunity to look up response options and may therefore not truly reflect awareness or real-life practice. As a self-reporting tool, responders may report the expected response rather than actual practice. With limited qualitative free text questions, reasoning behind GP responses is not possible to fully interpret.

### Comparisons with existing literature

Our findings identified some KAP items that may influence patient management, with some similarities to previously published primary care relevant data. However, given the diverse epidemiology and differences in delivery of primary care compared to the UK, the relevance of some of these similarities is unclear.^9,16,17,20–22,28,29^

Antibiotic prophylaxis as a preventative treatment, was a more common consideration in the England-based responder group and at a similar proportion as that identified in a physician survey in Connecticut.^29^ With limited evidence for prophylaxis in Europe and the UK, it is not currently recommended, even in UK regions with higher endemicity.^12^

Awareness of EM as pathognomonic for LD was significantly lower in the England-based responder group. Our findings show good recognition by Scotland-based responders, that is also suggested in recent data from a highly endemic Scottish region, with over 65% of LD cases having an EM relevant Read code, compared to <30% of LD cases from an earlier UK study.^5,9^ This may reflect an improvement in clinical diagnosis of LD with EM, although is also likely to be influenced through the addition of the ‘suspected’ LD and EM Read codes, introduced in 2014. This publication also identified doxycycline prescribing for shorter durations than recommended in NICE guidance in 27% of cases, concurring with 30% of our Scottish-based responders who indicated prescribing knowledge at non-NICE recommended dose and/or duration.^9^

Over 70% of England-based and 30% of Scotland-based responders would access diagnostic testing for a patient with EM, although the opportunity to capture whether this testing would result in treatment delay or influence diagnosis, was missed in our survey. These findings are also in common with published data where some contraindicated testing has been identified regardless of disease incidence, although this may decrease with increasing incidence or caseload.^16,17,20–22,28^ Inclusion of non-recommended serological testing in EM patient management, may reflect limited awareness of testing limitations in early infection, as well as clinician (or patient) frustration with diagnostic uncertainty.^14,29,30^

LD symptoms can be diverse, with clinical manifestations likely reflecting local prevalence of *B. burgdorferi* genospecies.^3^ Less well associated symptoms of AV block in both responder groups and cranial neuritis (with/without FNP) in the England-based responder group, concur with a KAP survey of Canadian primary care physicians KAP.^16^ EM manifestations associated with disseminated infection (multiple EM rashes and an EM rash away from a tick bite), may be more challenging to diagnose and were less well associated by both responder groups. Limited UK data is available regarding disseminated EM symptoms, although a Swedish study identified multiple EMs within PCR-confirmed EM cases at a higher frequency than expected.^31^ Within the UK, cardiac symptoms were documented in 1% and neurological symptoms in 16% of cases, with FNP commonly associated with paediatric LD.^32,33^ Improving GP awareness of these less familiar LD symptoms, may expediate appropriate referral and improve patient management.

### Implications for existing practice

Evolving tick habitat can include urban green spaces such as parks and private gardens, in addition to rural locations and clinician awareness of geographical risk will be helpful to elucidate exposure in some cases.^34–36^ Robust responder knowledge of commonly occurring LD signs and symptoms, may be enhanced through awareness of less familiar symptoms. Clinician confidence to clinically diagnose LD through recognition of EM, may help reduce associated contraindicated testing, preventing an unnecessary patient procedure and reducing laboratory testing burden and diagnostic uncertainty. Avoidable antibiotic therapy may occur due to misdiagnosing a tick bite reaction as EM, or from administered antibiotic prophylaxis. Standardising prescribing habits for symptomatic patients to NICE guidance, may help allay patient concerns regarding symptoms and sequelae associated with LD.

Clinician awareness of LD may be influenced through local incidence and relevant consultation rate, as well as public health initiatives at a regional, national, or UK level. Heterogeneity in awareness and practice is likely to be multifactorial and include GP perception of local risk and relevance to practice region.^37^ Initiatives such as the Lyme Disease General Practice Sentinel Scheme may be of future value to improve GP awareness, as well as standardising primary care data for future epidemiology studies.^8^

## Data

The dataset relied on in this article is available from the corresponding author on reasonable request.
